# A filtering approach for statistical inference in a stochastic SIR model with an application to Covid-19 data

**DOI:** 10.1093/biostatistics/kxaf036

**Published:** 2025-10-26

**Authors:** Katia Colaneri, Camilla Damian, Rüdiger Frey

**Affiliations:** Department of Economics and Finance, University of Rome Tor Vergata, Via Columbia 2, 00133, Rome, Italy; Department of Mathematics, Vrije Universiteit Amsterdam, De Boelelaan 1111, 1081 HV, Amsterdam, The Netherlands; Institute for Statistics and Mathematics, WU Vienna University of Economics and Business, Welthandelplatz, 1, 1020, Vienna, Austria

**Keywords:** stochastic SIR model, nested particle filtering, parameter inference, hidden Markov model, epidemiological data

## Abstract

In this paper, we consider a discrete-time stochastic SIR model, where the transmission rate and the number of infectious individuals are random and unobservable. This model accounts for random fluctuations in infectiousness and for non-detected infections. Thus, statistical inference has to be performed in a partial information setting. We adopt a Bayesian approach and use nested particle filtering to estimate the state of the system and the parameters. Moreover, we discuss forecasts and model tests based on the posterior predictive distribution. As a case study, we apply our methodology to Austrian Covid-19 infection data.

## 1. INTRODUCTION

Infectious diseases have key characteristics that simple epidemiological models may not fully capture. To begin with, there is randomness in the transmission process of a disease, particularly when the population size is small. This has given rise to the development of stochastic epidemic models [see, e.g. Andersson and Britton (2012) for further motivation]. Moreover, the infection or transmission rate may depend on several random factors, such as changes in the infectiousness of the virus, environmental conditions, seasonality, and, eventually, policy measures. Such considerations suggest that the rate should be modeled as a stochastic process. Finally, many infections go undetected because medical tests are often necessary to confirm a diagnosis. These features imply that an analyst cannot fully observe the components of the epidemiological system. In particular, the transmission rate and the true number of infections are latent.

To account for these features, we develop a stochastic and partially observable SIR model with a random transmission rate. In a first step we frame our model within the setting of a hidden Markov model (HMM). This consists of two components: a latent Markov process (the so-called state process), and an observable process (the so-called measurement or observation process) which is affected by the state, see Elliott et al. (2008) and Churchill (2005) for details. In our specific setup, the state process consists of the compartments of the SIR model and of the transmission rate. The observation process, on the other hand, is given by the number of newly confirmed cases. In a second step, we extend the framework to include quarantine measures. This is done by immediately removing the newly confirmed cases from the pool of infectious people. A further extension of the model with other compartments, such as vaccinations, is discussed in the online Supplementary Material (Colaneri et al. 2025).

Parameter estimation for this type of models is challenging due to noisy observations, as well as randomness and non-linearity in the transmission process. In this paper, we address the problem using a Bayesian approach to statistical inference. Our objective is to approximate the posterior distribution of the state variables and of the model parameters. To achieve this, we rely on sophisticated techniques from stochastic filtering and adapt the nested particle filter (NPF) of Crisan and Miguez (2018) to our setup. The ensuing algorithm is recursive, allowing new information to be efficiently incorporated. Simulation experiments, detailed in the online Supplementary Materials, see Colaneri et al. (2025), show that performance on simulated data is very satisfactory.

Our approach offers many advantages. First, its Bayesian nature makes it easily adaptable if additional sources of information—eg sewage data in our context—become available. In a similar spirit, expert opinions on the state of the epidemiological system are readily incorporated into prior distributions. Second, our methodology is ideally suited for forecasting of future infection numbers. This is an important application of epidemiological models, as these forecasts are used to gauge potential stress for the health system and to inform decisions on containment measures. In our context, forecasts are based on the *posterior predictive distribution*, so that parameter uncertainty is taken into account naturally, making the procedure robust. Moreover, recursiveness of the NPF allows us to easily update the predictive distribution to incorporate new information. Finally, our approach makes it possible to conduct formal goodness-of-fit tests for the posterior predictive distribution.

As a case study, we apply the version of our epidemiological model with quarantine to Covid-19 infection data from Austria. A comparison with official estimates of the Austrian health agency AGES shows that our approach produces qualitatively similar results for the *effective reproduction number*. Moreover, we discuss the forecasts generated by the model and compare them with realized infections.

The rest of the paper is organized as follows. In the next paragraph we survey the existing literature; Section 2 introduces the model; Section 3 describes the NPF; Section 4 discusses estimation results for Austrian infection data; Section 5 focuses on forecasts and model tests; Section 6 concludes.

*Literature review.* It is nowadays well established that randomness is a key feature in the development of epidemics. As explained in Andersson and Britton (2012), stochastic models are the natural way to describe the spread of a disease within a given population. Deterministic models, on the other hand, provide a reasonable approximation only under conditions where the law of large numbers applies [see also Allen (2008) and Britton et al. (2019) for further reference]. A stochastic epidemic model featuring random transmission rate and unobservable state variables is employed in Stocks et al. (2020) to describe the spread of Rotavirus infections in Germany. In that study the estimation problem is addressed using a frequentist methodology, specifically the *iterated filtering* approach of Ionides et al. (2015); see also King et al. (2016). Our model significantly differs from theirs in state dynamics—eg we include quarantine measures—and, most importantly, in the way randomness in the transmission rate is modeled. Moreover, we use a different estimation approach.

Other interesting contributions are Sun et al. (2015) and Corbella et al. (2022). Sun et al. (2015) study parameter estimation and provide a comparison of three partially observed dynamical models for the evolution of biological, ecological or environmental processes, using approximate Bayesian computing. In Corbella et al. (2022), an epidemiological system is described via a state-space model, where randomness is used to address relatively rare severe events. Parameter inference is conducted using a pseudo-marginal approach applied to multiple dependent datasets. Finally, Calvetti et al. (2021) and Cazelles et al. (2021) also use PMCMC algorithms to analyze Covid-19 data.

In incomplete information settings, several papers study epidemiological phenomena via the (extended) Kalman filter. For example, Narci et al. (2021) propose a Gaussian approximation of a Markovian jump model to describe an epidemic dynamics with constant transmission rate, and with compartments of susceptible and infectious that are unobservable. This method is applied to data on influenza in a British boarding school in 1978. Hasan et al. (2022) use the extended Kalman filter (EKF) within an SIR model with additional Gaussian noise to analyze Scandinavian Covid-19 data. However, their model exhibits somewhat implausible dynamics; for instance, the effective reproduction number is modeled as a random walk with Gaussian increments that can, therefore, become negative. Other studies, such as Sebbagh and Kechida (2022); Song et al. (2021), have considered a stochastic version of an SIR model with Gaussian noise to describe the spread of Covid-19 virus, using the EKF or the unscented particle filter [see, eg Papageorgiou and Tsaklidis (2023)]. Nevertheless, it is important to stress that the SIR model forms a non-linear system. The EKF linearizes these dynamics in a somewhat ad-hoc fashion, so that optimality and stability of the estimates cannot be guaranteed [see eg Budhiraja et al. (2007)]. Despite these limitations, the EKF remains widely used due to its simplicity and the fact that it works well when non-linearities are small.

## 2. MODEL SPECIFICATION

In the sequel, we introduce a stochastic version of the standard SIR model, where both the actual number of infected people and the infection rate are random and unobservable. Since we will test our methodology on Covid-19 data, our model specifications account for some special features of this context, such as testing and quarantine regulations. We describe the model in two steps. In the first step (Section 2.1), we consider a simpler case in which quarantine is not taken into account, and develop a discrete-time HMM for the epidemiological system. In the second step, we include quarantine. This generates an additional dependence channel between the state variable and the observations that we explain better in Section 2.2.

*Notation.* We begin by introducing key variables of the model. First, since infection numbers are usually reported on a daily or a weekly basis, we work in a discrete-time setting with time points $ (0\,=\,t_{0},t_{1},\dots, t_{n},\dots) $ (in the data analysis, we assume that $ t_{n}-t_{n-1} $ is 1 d). We consider a population with $ N $ individuals and we assume, for simplicity, that the population size stays constant over time. This assumption approximates the case where the observation period is short and the number of deaths due to infections is small compared to the population size. We then let

•
$ S_{n} $ be the number of susceptible individuals at time $ t_{n} $;
•
$ I_{n} $ be the number of infectious persons at time $ t_{n} $ who can generate new infections in the next period, ie $ [t_{n},t_{n+1}) $;
•
$ I_{n}^{+} $ be the number of individuals who get infected in $ [t_{n},t_{n+1}) $;
•
$ I_{n}^{-} $ be the number of individuals who were infectious at $ t_{n} $ but are removed from the number of infectious people at time $ t_{n+1} $, for instance since they recovered or, for certain diseases, are in quarantine;
•
$ R_{n} $ be the number of so-called *removed* individuals; that is, people who are either immune or in quarantine at time $ t_{n} $;
•
$ P_{n} $ be the number of newly *reported* infections (such as positive tests) at time $ t_{n} $, where we may assume that testing starts at $ t_{1} $;
•
$ \beta_{n} $ be the *infection* or *transmission* rate, that is the average number of people that are infected in $ [t_{n},t_{n+1}) $ by one infectious person in a population where everyone is susceptible; see equation (2.2).

A process is indicated by capital letters without time index, eg $ S $ denotes the discrete-time process $ (S_{n})_{n\,=\,0,1 , \dots} $. We will also use the notation $ P_{1: n} $ to indicate the history of the process up to $ n $, that is the sequence of random variables $ P_{1},\dots, P_{n} $. Finally, we adopt the usual convention that upper case letters are random variables and lower case variables are data points or samples.

### 2.1. A HMM for epidemics without quarantine

In what follows, we deal with a set of variables, called the *stock* variables, that represent the number of people in each compartment at time $ t_{n} $, given by $ (S_{n},I_{n},R_{n}) $. These, together with the transmission rate $ \beta_{n} $, form the unobservable state. For convenience we let $ \Psi_{n}=\log(\beta_{n}) $ and model the dynamics of the process $ \Psi_{n} $. There is also a set of so-called *flow* variables, given by $ (I^{+}_{n},I^{-}_{n}) $ and $ P_{n} $, that are used to represent the changes in the stock variables from $ t_{n} $ to $ t_{n\,+\,1} $. Among flow variables, $ I^{+}_{n} $ and $ I^{-}_{n} $ are latent, whereas $ P_{n} $ provides the observation.

*Dynamics of the state variables.* Next, we describe the evolution of the state of the system, namely the dynamics of processes $ (S, I, R) $ and $ \Psi $. Throughout, we fix a distribution for $ I_{0} $, $ R_{0} $ and $ \Psi_{0} $. We begin with the logarithmic transmission rate. We assume that $ \Psi $ follows a first-order autoregressive process given by


(2.1) \begin{align*}\Psi_{n}=\Psi_{n-1}+\kappa(\mu-\Psi_{n-1})+\sigma Z_{n-1}\,,\quad n=1,2 , \dots\end{align*}

for a sequence of independent standard normal random variables $ \{Z_{n}\}_{n\,=\,0,1 , \dots} $ and parameters $ \kappa , \sigma > 0 $ and $ \mu\in\mathbb{R} $.

Next, we introduce the dynamics of $ S $, $ I $ and $ R $. By definition, the number of susceptible people satisfies $ S_{n}=N-I_{n}-R_{n} $, for $ n\,=\,0,1 , \dots $, so that $ S_{n} $ can be identified from $ I_{n} $ and $ R_{n} $ (for this reason, we can omit $ S_{n} $ in the set of state variables). The process $ I $ evolves according to $ I_{n}=I_{n-1}+I^{+}_{n-1}-I^{-}_{n-1},\quad n\,=\,1,2 , \dots $. The new infections $ I_{n}^{+} $ are modeled as a Poisson random variable,


(2.2) \begin{align*} I_{n}^{+}\sim{\rm Poisson}(\lambda_{n})\;\text{with }\lambda_{n}=\beta_{n}I_{n}\frac{S_{n}}{N},\quad n=0,1 , \dots , \end{align*}

The model claims that the expected number of new infections is proportional to the number of infectious people and to the fraction of susceptible individuals in the whole population.

Remark 2.1 We now briefly comment on our assumptions on the transmission model.

(i) It is natural to assume that, for small $ t_{n+1}-t_{n} $, the quantity $ \beta_{n}\frac{I_{n}}{N} $ is small. In that case, we can interpret it as the probability that a susceptible person at time $ t_{n} $ gets infected over the interval $ [t_{n},t_{n+1}) $. If, moreover, infection events are assumed to be independent across susceptible individuals, and the susceptible population $ S_{n} $ is large, then we can use the Poisson approximation to justify the model (2.2).
(ii) To motivate the assumption for the infection rate dynamics (2.1), we observe that this process is stationary and mean-reverting around the value $ \mu $, which is a common behavior for both endemic and pandemic diseases. In fact, the infection rate of an endemic disease is stationary by definition, whereas, for pandemics, stationarity is often enforced by policy measures. For instance, in the pandemic phase of Covid-19, many European governments tightened containment rules in periods of high infection numbers (corresponding to high values of the reproduction number $ \mathcal{R}_{n} $, see below) and loosened measures after infection numbers had fallen to more sustainable levels.

Infectious people who are not detected move to the removed state upon recovery from the infection. Thus, at the end of the time interval $ [t_{n},t_{n\,+\,1}) $, the number of infectious people is reduced by $ I_{n}^{-}=\gamma \! I_{n} $, for $ n\,=\,0,1,2 , \dots $, where $ \gamma > 0 $ is the inverse of the average time a non-detected individual stays infectious. Finally, we assume that $ R_{n}=R_{n-1}+I_{n-1}^{-}-\delta R_{n-1} $, for $ n\,=\,1,2 , \dots $, where the parameter $ \delta > 0 $ is such that $ 1/\delta $ is the average time an infected person enjoys immunity. In other words, a removed person loses immunity and becomes susceptible again at rate $ \delta $. For instance, $ \delta\sim\frac{1}{200} $ says that people who recovered from the virus on average do not get infected again for about 200 time units (ie days, in our case). Summarizing, the dynamics of the system are as follows. For $ n\,=\,1,2 , \dots $,


(2.3) \begin{align*}\left\{\begin{aligned}\Psi_{n}&=\Psi_{n-1}+\kappa(\mu-\Psi_{n-1})+\sigma Z_{n-1}\nonumber\\ I_{n}&=I_{n-1}+I_{n-1}^{+}-I^{-}_{n-1}\nonumber\\ R_{n}&=R_{n-1}+I_{n-1}^{-}-\delta R_{n-1}\,,\end{aligned}\right.\end{align*}

where $ I_{n}^{+}\sim{\rm Poisson}\big(\beta_{n}\frac{I_{n}}{N}S_{n}\big) $, $ I_{n}^{-}=\gamma \! I_{n} $ and $ Z_{n}\sim N(0,1) $. From (2.3) and the one-to-one correspondence between $ \beta_{n} $ and $ \Psi_{n} $ we get that the distribution of the triple $ (I_{n},R_{n},\beta_{n}) $ can be described in terms of a transition kernel that depends only on $ (I_{n-1},R_{n-1},\beta_{n-1}) $, therefore forming a discrete-time Markov chain.

*The observations.* The true number of infectious people is unknown, as this quantity includes asymptomatic infections or infected individuals who have not (yet) taken a test. Moreover, since infections are random and unobservable, we cannot observe the infection rate $ \beta_{n} $. At any time $ n\,=\,1,2 , \dots $, the available information is thus provided by the number of newly reported cases $ P_{n} $, whereas all other state variables are latent. To describe the dynamics of new cases, we assume that an infectious person at time $ t_{n} $ is detected with probability $ q\in[0,1] $. The parameter $ q $ accounts for the availability and reliability of tests and/or for the intensity of public screening programs. We assume that testing occurs independently across infected people and starts at time $ t_{1} $. Hence, the conditional distribution of positive tests at time $ t_{n} $, given the number $ I_{n} $ of infectious people at time $ t_{n} $, is binomial with parameters $ I_{n} $ and $ q $. That is to say, $ P_{n}\sim{\rm Binomial}\left(\left\lfloor I_{n}\right\rfloor, q\right)\!, n\,=\,1,2 , \dots $, where $ \left\lfloor\cdot\right\rfloor $ denotes the floor function. Formally, at any time $ t_{n} $, the available information can be described by the history $ P_{1: n} $.

Note that other data sources, such as results from sewage screening or sentinel systems, are easily integrated into our approach, provided that the conditional distribution of these variables given $ I_{n} $ is known (however, this is left for future research).

### 2.2. An extension with quarantine

In this section, we consider an extension of the model allowing for quarantine measures. This, in turn, implies a modification in the dynamics of removed individuals; that is, we assume that a person who tests positive is immediately removed from the pool of infectious people to reflect quarantine measures or self-isolation precautions. Infectious people who are not detected move to the removed state upon recovery from the infection, as before. Thus, for any $ n\,=\,0,1,2 , \dots $, the number of infectious people is reduced by $ I_{n}^{-}=P_{n}+\gamma \!I_{n} $, where we set $ P_{0}=0 $ if it is assumed that testing starts at time $ t_{1} $. Note that we use the same notation $ I_{n}^{-} $ to indicate the number of individuals who are removed from the pool of infectious people. In summary, the dynamics of the system are as follows: for $ n\,=\,1,2 , \dots $,


(2.4) \begin{align*}\left\{\begin{aligned}\Psi_{n}&=\Psi_{n-1}+\kappa(\mu-\Psi_{n-1})+\sigma Z_{n-1}\nonumber\\ I_{n}&=I_{n-1}+I_{n-1}^{+}-I^{-}_{n-1}\nonumber\\ R_{n}&=R_{n-1}+I_{n-1}^{-}-\delta R_{n-1}\,,\end{aligned}\right.\end{align*}

where $ I_{n}^{+}\sim{\rm Poisson}\big(\beta_{n}\frac{I_{n}}{N}S_{n}\big) $, $ I_{n}^{-}=P_{n}+\gamma \!I_{n}, $  $ P_{n}\sim{\rm Binomial}\left(\left\lfloor I_{n}\right\rfloor, q\right) $ and $ Z_{n}\sim N(0,1) $. As before, the observations are the history of confirmed cases, $ P_{1: n} $ for $ n\,=\,1,2 , \dots $.

**Fig. 1.**
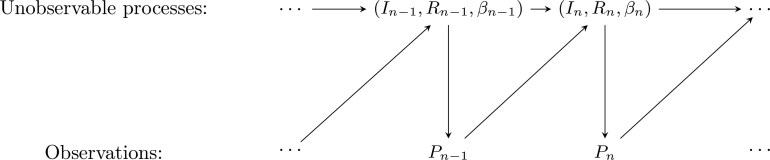
Illustrative description of the state-observation model: the case with quarantine.

The evolution of the state variables in the model with quarantine depends on the observations. Hence, this model no longer falls into the class of standard HMMs. This is illustrated schematically in Fig. 1, where we see that the observation $ P_{n} $ is determined by the state at time $ t_{n} $ (via $ I_{n} $) *and* affects the state variables at time $ t_{n\,+\,1} $ (via $ R_{n\,+\,1} $). Nonetheless, the model with quarantine is amenable to particle filtering as explained in Section 3.

*Reproduction number.* The effective reproduction number $ \mathcal{R}_{n} $ is an index that measures the number of individuals who are infected, on average, by a specific infectious person, given the state of the pandemic system at time $ t_{n} $. To identify $ \mathcal{R}_{n} $ in the model with quarantine (for the version of the model without quarantine it is known that $ \mathcal{R}_{n}\approx\frac{\beta_{n}}{\gamma}\frac{S_{n}}{N} $), note first that an infected individual transmits the disease, on average, to $ \beta_{n}S_{n}/N $ people per day. Moreover, the time that elapses before an infected person transits to the compartment of removed individuals, ie to $ R $, is the minimum of $ \tau^{\mathrm{rec}} $ (the time up to recovery) and $ \tau^{\mathrm{quar}} $ (the time until the person tests positively and is put into quarantine). Under our model dynamics, $ \tau^{\mathrm{rec}} $ and $ \tau^{\mathrm{quar}} $ have a geometric distribution with parameter $ \gamma $ and $ q $, respectively. Moreover they are independent, so that $ \min\{\tau^{\mathrm{rec}},\tau^{\mathrm{quar}}\} $ follows a geometric distribution with parameter $ \gamma+q-\gamma \! q $. Hence, the expected time up to removal satisfies $ \mathbb{E}(\min\{\tau^{\mathrm{rec}},\tau^{\mathrm{quar}}\})=(\gamma+q-\gamma \! q)^{-1}\, $ and


(2.5) \begin{align*}\mathcal{R}_{n}=\frac{\beta_{n}}{\gamma+q-\gamma \! q}\frac{S_{n}}{N}\,.\end{align*}

## 3. STATISTICAL METHODOLOGY

When discussing statistical inference for the model described in Section 2, we can distinguish two problems: the *filtering* problem and the *parameter estimation* problem. The filtering problem is concerned with inferring the state process—in our case, the triple $ (\beta, I, R) $—conditional on the available observations and on a given parameter vector $ \boldsymbol{\theta} $. The parameter estimation problem, on the other hand, is concerned with the case in which parameters values are also unknown. A straightforward way to estimate state and parameters jointly within a bootstrap filter would be to augment the state space so that it includes a parameter vector as a constant-in-time variable; however, this implies that the parameter space is explored only in the initialization step of the algorithm, making it destined to degenerate, see eg Kantas et al. (2015). It is thus important to use a methodology that periodically reintroduces diversity in the parameter set.

With this in mind, here we chose to adapt the NPF algorithm of Crisan and Miguez (2018). Under the assumption that the parameter space is a compact set and the conditional probability distribution of the observations is bounded uniformly over that set, this algorithm allows us to track the posterior distribution of the (static) unknown parameters in our model, as well as the joint posterior distribution of parameter and state variables, in a recursive fashion. More specifically, the NPF algorithm consists of two nested layers of particle filters: an “outer” filter, which approximates the posterior of $ \boldsymbol{\theta} $ given the observations, and a set of “inner” standard bootstrap filters [see eg Gordon et al. (1993)], each corresponding to a sample generated in the outer layer and yielding an approximation of the posterior measure of the state, conditional on both the observations and the given sample of $ \boldsymbol{\theta} $. In particular, in each algorithm iteration, the parameter particles are first agitated, or *jittered*, using a truncated Gaussian kernel with mean corresponding to the parameter estimates at the previous time point and fixed variance. In this way, all particles are subjected to a small perturbation, restoring their diversity.

The jittering step is key to make the algorithm recursive, unlike other popular algorithms, such as iterated filtering and some other particle Markov chain Monte Carlo (PMCMC). Moreover this feature makes our approach particularly suitable for forecasting, see Section 5. A description of the algorithm, including the corresponding pseudocode and further computational details, is available in the online Supplementary Material, see Colaneri et al. (2025).

*Parameter estimation via nested particle filtering.* In our setup, we can distinguish two sets of parameters influencing the epidemiological system. On the one hand, we have the triple $ \boldsymbol{\theta}=(\kappa , \sigma , \mu) $, which governs the dynamics of the infection rate. On the other hand, we have the rates $ q $, $ \gamma $ and $ \delta $. It is worth noting that our observation process consists only of the number of reported cases and that, due to such limitations in the nature and in the length of the observation time series, we will not be able to estimate all model parameters with reasonable accuracy and we will thus resort to fixing the rates $ q , \gamma $ and $ \delta $ exogenously. That is, we assume that the parameters $ q $, $ \gamma $ and $ \delta $ are derived from other data (for instance, different medical data, sewage data, as well as the results of other statistical studies) and hence represent a fixed input of our model. As an example of a study reporting calibrated detection rates for Austria specifically, we refer the reader to Rippinger et al. (2021). A formal analysis of this issue is given in the Supplementary Material of Stocks et al. (2020). They show that, for a deterministic SIR model in its endemic (stationary) state, only the infection rate $ \beta $ can be estimated from infection data, while the other SIR parameters have to be estimated from other data sources.

*Analysis on simulated data.* The online Supplementary Material provides an extended analysis of the NPF algorithm performance on simulated data for the model with quarantine. This analysis shows that the algorithm captures the effective reproduction rate quite well. Moreover, it indicates that parameter $ \mu $ is easier to estimate compared to $ \kappa $ and $ \sigma $. We have also conducted a comparative analysis of our methodology with the iterated filtering (IF) approach described in Ionides et al. (2015). Parameter estimates are roughly similar. However, applying the IF requires a careful selection of hyperparameters, as the performance of the algorithm is very sensitive to these choices. We refer to Colaneri et al. (2025) for the details.

## 4. EMPIRICAL RESULTS

**Fig. 2.**
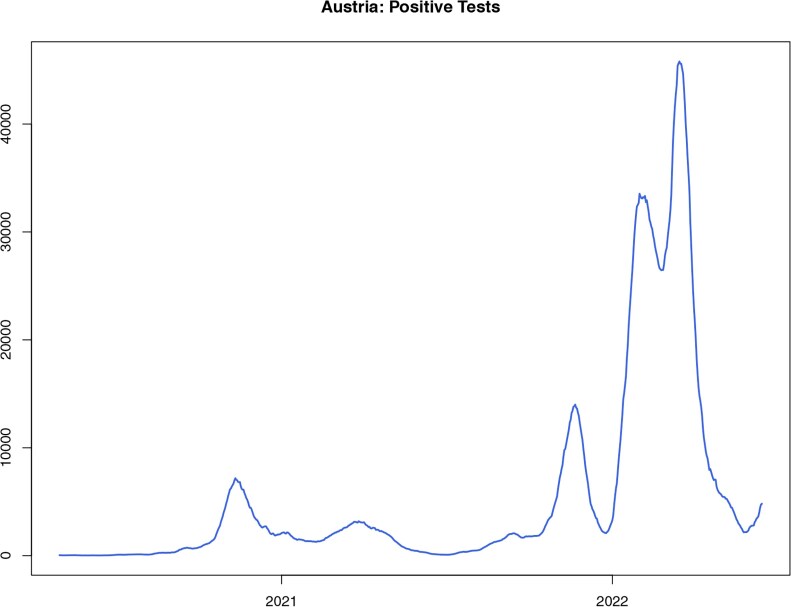
Confirmed cases of Covid-19 in Austria (from 1 May 2020 to 15 June 2022).

In this section, we apply the NPF approach to Austrian Covid-19 data from 1 May 2020 to 15 June 2022. The data used for this analysis were publicly available from the AGES website https://covid19-dashboard.ages.at/. We do not include further data, as this would mean to consider earlier and later periods of the pandemic in which policy measures to contain the virus and testing behavior of the Austrian population were substantially different, for example, because of very limited testing before May 2020, or the reduction of quarantine time after 15 June 2022. We also specify that in this analysis we use the version of the model with quarantine described by Equations (2.4). Figure 2 shows the positive tests recorded over these 2 yrs. We use a 7-d rolling average of confirmed cases to avoid weekly seasonality effects, such as the fewer tests performed over the weekend. We set the exogenous parameters according to the following values: $ q\,=\,10\% $, $ \gamma\,=\,1/10 $, $ \delta\,=\,1/200\,=\,0.05 $; number of days is $ N^{days}=776 $ (including time 0), the time step $ \Delta=t_{n}-t_{n-1}=1 $ day, and finally the total number of individuals is $ N\,=\,9.028\cdot 10^{6} $, corresponding to Austrian population. The hyperparameters of the particle filter algorithm are given in Appendix B of the Supplementary Material [see Colaneri et al. (2025)]. Hyperparameters, such as the variance of the jittering kernel and the number of particles, have been fine-tuned through preliminary simulation experiments, taking also into account the theoretical convergence results from Crisan and Miguez (2018). However, no significant difference has been observed in parameter estimation for reasonable values of such quantities.

**Fig. 3.**
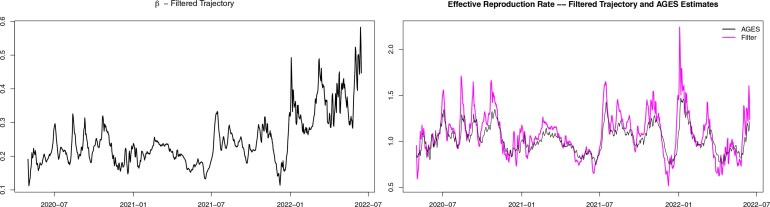
Left: Estimates for the infection rate $ \beta $ from Austrian Covid-19 data (from 1 May 2020 to 15 June 2022). Right: Estimates for the effective reproduction number from Austrian Covid-19 data (from 1 May 2020 to 15 June 2022): in magenta the filtered estimate of $ \mathcal{R}_{n} $ using our methodology; in black the estimate published by the Austrian health agency AGES. The authors recommend that readers consult the colour version of the figure available in the electronic article.

We begin with the filtered estimate of the infection rate $ (\beta_{n})_{n\geq 1} $, which is provided in the left panel of Fig. 3. Here, we observe an upward trend from the beginning of 2022, which is most likely due to the arrival of a highly contagious virus variant (the Omicron variant).

Now, we focus on the effective reproduction number $ (\mathcal{R}_{n})_{n\geq 1} $, see equation (2.5). In the right panel of Fig. 3, we compare our filtered estimates (in magenta) with the official estimate published by the Austrian health agency AGES (in black). The latter is computed using a renewal modeling approach; in particular, a Bayesian model with Gamma-distributed prior for $ \beta $ and Poisson observations, see Richter et al. (2020) for details and see also the Supplementary Material of Cori et al. (2013) for a description of the methodology. The plot shows that the qualitative behavior of both estimates is very similar; however, our filtered estimate displays more variability and more pronounced spikes, particularly starting from mid-2021. While it is not possible to judge with certainty which of the two estimates is closer to the “true” value of $ \mathcal{R}_{n} $, when dealing with real data, evidence form the simulation study included in the Supplementary Material [see Colaneri et al. (2025)], shows that the estimated reproduction number exhibits a less volatile pattern than the true trajectory of $ \mathcal{R}_{n} $. This suggests that the higher variability of our estimates for $ \mathcal{R}_{n} $ compared to the AGES estimate may in fact be an advantage.

Note that, while the infection rate $ \beta $ in the first half of 2022 is persistently higher than in 2021 (see the left panel of Fig. 3), the effective reproduction number displays a spike at the beginning of 2022 and then immediately settles again around one (see the right panel of Fig. 3). This is due to the counteracting effect that a large part of the population got infected in a small time window due to higher contagiousness of the Omicron variant, which reduced the number of susceptible individuals substantially.

**Fig. 4.**
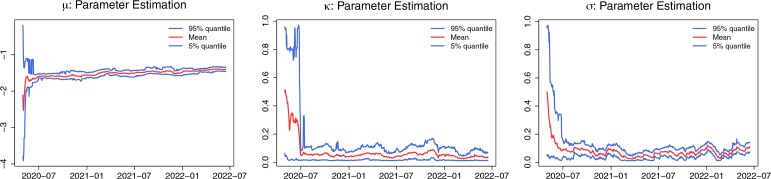
Posterior estimates for $ \mu $, $ \kappa $, and $ \sigma $. The two blue lines to the 5%- and 95%-quantile of the posterior distribution and the red line to its mean. The authors recommend that readers consult the colour version of the figure available in the electronic article.

Next we discuss parameter estimates for $ \boldsymbol{\theta}=(\kappa , \sigma , \mu) $. Figure 4 shows how, over time, the posterior distribution of $ \boldsymbol{\theta} $ gets more and more concentrated. In line with the results from the simulation study [see the Supplementary Material Colaneri et al. (2025)], we observe that the posterior-mean estimate of the parameter $ \mu $ fluctuates the least. Notice that, from the beginning of the second year of data, the posterior-mean estimate of $ \mu $ seems to increase. This effect may suggest that parameters are, in reality, time-varying (in particular, the arrival of new virus variants might have started a new regime). However, we need to be careful with such conclusions, as our simulation study revealed the limitations in the accuracy of estimates obtained using a relatively short observation series. One possible way to investigate our conjecture is to split the data into two periods—each potentially corresponding to a different regime—and perform parameter inference separately in both to compare estimates. This would however reduce the amount of data available for each regime, hence potentially compromising estimation accuracy. For this reason, we do not investigate our conjecture further.

## 5. FORECASTING AND MODEL TESTS

A relevant application of an epidemiological model is to make forecasts regarding the development of infection numbers, which are used to gauge potential stress for the health system and which serve as a basis for decisions on containment measures. Moreover, analyzing the quality of model-based predictions represents a natural way of testing a given model. Therefore, in this section, we discuss forecasts and model tests for our setup.

### 5.1. Methodology

The key quantity for forecasting and testing is the *posterior predictive distribution* of future positive tests over a time horizon $ \Delta $, with distribution function $ {F}_{n , \Delta}(y)=\mathbb{P}(P_{n+\Delta}\leq y\mid P_{1: n})\, $. To compute an estimate $ \widehat{F}_{n , \Delta} $ of $ {F}_{n , \Delta} $, we rely on simulations, using the following schematic algorithm.

1. Run the NPF over the period $ [0, t_{n}] $; the output $ (\theta_{n}^{(k)},x^{(k, m)}_{n}),\ k=1 , \dots, K , \ m=1 , \dots, M $, with $ x^{(k, m)}_{n}=(i^{(k, m)}_{n},r^{(k, m)}_{n},\psi^{(k, m)}_{n}) $, provides an approximation of the conditional distribution of the model parameters and the state variables given $ P_{1: n} $.
2. To generate a realization from $ {F}_{n , \Delta}(x) $ draw an index $ (\hat{k},\hat{m}) $ at random and use $ (\theta_{n}^{(\hat{k})},x^{(\hat{k},\hat{m})}_{n}) $ to forward-generate trajectories of $ I, R , \psi $ and $ P $ over the horizon $ t_{n},\dots, t_{n}+\Delta $ using the dynamics (2.3) and (2.1).

Various point forecasts such as quantiles can be computed from the predictive distribution in a straightforward way. We would like to emphasize again that using a recursive method such as NPF allows to update the posterior estimate of Step 1. in an online fashion, which is very convenient if forecasts have to be redone when new information becomes available.

We fix some horizon $ \Delta $ and consider non-overlapping prediction dates $ t_{n_{1}},t_{n_{2}},\dots, t_{n_{\ell}} $, where $ t_{n_{j}}=t_{n_{1}}+j\Delta $. Then formal *statistical tests* of our methodology can be based on the following classical result of Rosenblatt (1952): if the predictive distribution is correctly specified, that is $ \widehat{F}_{n_{j},\Delta}={F}_{n_{j},\Delta} $ for all $ j $ (this is the null hypothesis for our model test), then, the random variables $ \widehat{U}_{j}:=\widehat{F}_{n_{j}}(P_{n_{j}+\Delta}) $, $ 1\leq j\leq\ell $, are independent and identically distributed standard uniform. Strictly speaking, this is true only if $ \widehat{F}_{n , \Delta} $ is continuous. In our setup $ P_{n} $ is conditionally binomial and $ \widehat{F}_{n , \Delta} $ is computed by simulation, therefore it is discrete. However, the number of simulations used is large and the conditional distribution of $ P_{n} $ is very well approximated by a normal, so that under the null hypothesis the distribution of $ \widehat{F}_{n , \Delta}(P_{n+\Delta}) $ is very close to a standard uniform distribution. The result of Rosenblatt (1952) is the basis for a multitude of statistical tests, see for instance Gordy and McNeil (2020). Simple tests use *quantile exceedances*. Fix $ \alpha\in[0,1] $, then the sequence of quantile exceedances $ I_{n_{j}}^{\alpha}=1_{\{P_{n_{j}+\Delta} > {q}_{\alpha}(\widehat{F}_{n_{j},\Delta})\}} $, $ 1\leq j\leq\ell $, consists of independent and identically Bernoulli-distributed random variables with $ p\,=\,1-\alpha $. That implies that the number of exceedances $ M^{\alpha}=\sum_{j\,=\,1}^{\ell}I_{n_{j}}^{\alpha} $ has a binomial distribution with parameters $ \ell $ and $ p\,=\,1-\alpha $, which can be tested with a simple binomial test. More generally, one may test several quantile exceedances jointly by means of *multinomial tests* [see also Kratz et al. (2018) for details].

### 5.2. Empirical results

**Fig. 5.**
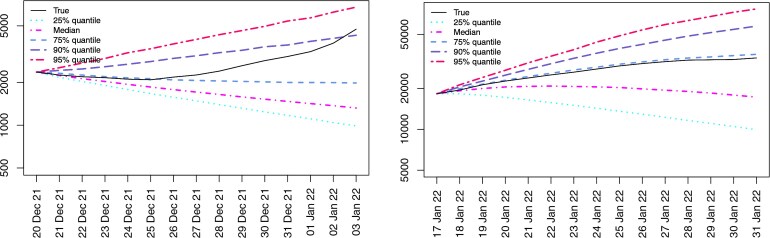
Left: quantiles $ q_{\alpha}(\widehat{F}_{n, \Delta}) $, $ \alpha\,=\,0.25,0.5,0.75,0.9,0.95 $, of $ \widehat{F}_{n, \Delta} $ for the prediction date $ t_{n}=20/12/2021 $ and prediction horizon $ \Delta $ between one and 14 days together with the observations; right: quantiles and mean of $ \widehat{F}_{n, \Delta} $ and realized positive tests for $ t_{n}=17/01/2022 $. Note that due to the skewness of the predictive distribution a logarithmic scale is used for the $ y $ axis.

We now apply these ideas to the Austrian Covid-19 data. We consider horizons up to 14 days, as this is a common forecasting horizon [prediction horizons of one and 2 wks are also taken, eg in Bicher et al. (2022)].

*Predictive distribution.* In Fig. 5, we plot several quantiles of $ \widehat{F}_{n , \Delta} $ for $ \Delta\,=\,1,2 , \dots , 14 $, together with the actually observed positive tests for two different prediction dates $ t_{n} $. The left plot shows the forecasts made on 20 December 2021—that is, shortly before the start of the wave of infection caused by the Omicron variant of the virus in Austria—while the right plot shows the forecasts from 17 January 2022. Note that the predictive distribution is very skewed, as can be seen from Table 1, where we report numerical values of various quantiles and of the mean of $ \widehat{F}_{n_{j},14} $ for these two prediction dates. This suggests that for many applications quantiles might be more suitable point forecasts than the mean.

**Table 1.** Observations, quantiles and mean of the predictive distribution $ \widehat{F}_{n , 14} $, for two different prediction dates and a horizon of 14 days.

Prediction date	$ P $	25%	50%	75%	90%	95%	Mean
20 December 2021	4,736	989	1,316	1,960	4,257	6,581	4,412
17 January 2022	33,543	10,076	18,112	35,658	57,613	82,352	29,782

Note that the distribution is very skewed with high upper quantiles and a mean exceeding the 90% quantile for 20 December 2021.

Looking at the plot on the left, we see that the model predicts well the decline of the wave of infection generated by the Delta variant of the virus, but the high infection numbers caused by the onset of Omicron variant of the virus reaches the 90% quantile of the predictive distribution toward the end of the forecast period. This is to be expected: since our model is informed only by observations of past positive tests, it is not “aware of” the emergence of a new virus variant. Such information would need to be entered manually by the epidemiologist, for instance as an artificial upward shift in the distribution of the infection rate at $ t_{n} $. The right plot shows that by 17 January 2022, the model has learned the different regime and actual cases are between the median and the 75% quantile of $ \widehat{F}_{n , \Delta} $. However, even on December 20, the infection numbers of the Omicron wave are below the 90% quantile of the predictive distribution, for most of the time and thus well within the range of possible future scenarios generated by our model.

We conclude the section by discussing quantile exceedances. We use a horizon of $ \Delta\,=\,14 $. Since posterior-mean estimates resulting from the NPF algorithm start stabilizing after a little more than a year, we chose 7 June 2021, as the date of the first test, and consider $ \ell\,=\,20 $ non-overlapping testing dates. We consider the quantile levels $ \alpha_{1}=0.25 $, $ \alpha_{2}=0.5 $, $ \alpha_{3}=0.75 $, $ \alpha_{4}=0.9 $ and $ \alpha_{5}=0.95 $ in our tests. In Table 2, we report the expected and the observed number of quantile exceedances, and see that in the upper tail they are quite close to their expected value. Note, however, that the precise outcome of numerical tests varies somewhat with the chosen quantile levels and testing horizon, in particular given the low number of available testing dates, which is why we are not presenting formal test results.

**Table 2.** Expected and observed quantile exceedances.

$ \alpha $	0.25	0.5	0.75	0.9	0.95
Exp.	15	10	5	2	1
Obs.	19	17	12	2	0

## 6. CONCLUSION AND DISCUSSION

*Summary.* In this paper, we proposed a discrete-time stochastic variant of the SIR model to describe the spreading of an infectious disease with unobservable random transmission rate and compartments. Observations are given by newly confirmed cases, such as positive tests. This structure reflects real-world epidemic dynamics more closely than classical deterministic models, particularly in settings with limited or delayed observations.

*Methodological contributions.* We focused on state and parameter estimation using the NPF approach. A key advantage of this method is its recursive nature, which allows to incorporate efficiently new information for updating the posterior distribution, as it becomes available. This feature makes the NPF particularly useful when dealing with prediction, and represents a major benefit of the NPF over competitor algorithms, like iterated filtering, which requires reprocessing the entire dataset as new information arrives, thereby making, eg the forecasting more involved. Another strength of the NPF is its ability to detect change points in model parameters, such as those triggered by the emergence of new virus variants, provided these changes occur relatively infrequently. We briefly illustrated this capability in our discussion of the estimates for the parameter $ \mu $ (see Fig. 4). However, a comprehensive investigation of this aspect is left for future work. On the other hand, parameters that exhibit rapid or continuous variation over time cannot be accurately estimated with our approach, as the NPF is not designed to handle high-frequency parameter shifts.

*Limitations and potential extensions.* Several simplifications were adopted in our model for clarity and tractability, which should be kept in mind in the interpretation of our results. For instance, transitions between compartments may lead to non-integer values. While this choice facilitates exposition and does not impact results in large populations, it could be refined by modeling transitions as Poisson processes, especially in applications involving small populations. We also chose not to include additional compartments such as vaccination or death in the main model. Our focus was to highlight the effects of random transmission rates and undetected infections. Nevertheless, the model can be extended to incorporate such compartments as needed.

Given the relevance of vaccinations in the management of Covid-19, in the online Supplementary Material (Colaneri et al. 2025) we discuss a theoretical extension of the model with vaccinations. This model falls into the class of *leaky vaccine models*, where vaccinated individuals have a reduced probability of infection upon each exposure to the virus. Leaky vaccine models realistically capture individual-level protection, particularly in the case of Covid-19, where vaccines have been shown to reduce susceptibility and severity but not to fully prevent infection, especially with variants like Omicron. However, at the aggregate level, our base model is already rich enough to capture the main features of the infection dynamics of the Covid-19 pandemic. In fact, the leakiness of Covid-19 vaccines—as opposed to all-or-nothing vaccines—suggests that their effect on the pandemic dynamics may be indirectly captured by introducing randomness in the transmission rate, as done in this paper. Thus, we chose to prioritize a simpler and more parsimonious specification for empirical analysis, and we leave a detailed empirical investigation of the extended model for future work.

## 7. REPLICATION PACKAGE

For replication purposes, we provide both the code and the data used in our empirical analysis as an R package, which can be installed directly from our GitHub repository via devtools::install_github(“camilladamian/stochSIRreplication”). Following the instructions available here, the plots reported above can be reproduced. A detailed description of the arguments of the various R functions, together with their default values, is available in the corresponding documentation (eg by running ?”stochSIR_NPF”). The default parameter choices follow those reported in Appendix C of Colaneri et al. (2025).

## Supplementary Material

kxaf036_Supplementary_Data
