# Supplementary material for: A filtering approach for statistical inference in a stochastic SIR model with an application to Covid-19 data
Source: Biostatistics. 2025 Oct 26;26(1):kxaf036. doi: 10.1093/biostatistics/kxaf036 (PMC12554006; doi:10.1093/biostatistics/kxaf036)
Supplement: kxaf036_Supplementary_Data [file kxaf036_supplementary_data.pdf]

Biostatistics (2025), **0**, 0, *pp.* **??–??**
doi:10.1093/biostatistics/output

A filtering approach for statistical inference in a stochastic SIR model with an application to Covid-19 data: Supplementary material

Katia Colaneri[1,*], Camilla Damian[2], Rüdiger Frey[3]

[1] *Department of Economics and Finance, University of Rome Tor Vergata.*

[2] *Department of Mathematics, Vrije Universiteit Amsterdam.*

[3] *Institute for Statistics and Mathematics, WU Vienna University of Economics and Business.*

1. Introduction

The supplementary materials in this note provide a comprehensive account of the statistical methodology employed in our study, a detailed simulation study and additional insights on the nested particle filtering algorithm, which complement the main analysis presented in Colaneri *and others* (2025). Furthermore, we include a comparison of our methodology with the iterated filtering method of Ionides *and others* (2015), which is frequently employed for hidden Markov models in epidemiology. This comparison highlights strengths, performance, and applicability of the two approaches, offering a deeper understanding of the advantages and trade-offs in our proposed methodology.

2. Model specification

For the convenience of the reader we recall the dynamics of the partially observable stochastic SIR model described in Section 2.2 of Colaneri *and others* (2025). Key features of that model are randomness and unobservability of the true number of infected individuals and of the infection rate. To illustrate the flexibility of our methodology, we moreover discuss an extension of the model that accounts for the possibility of vaccination. This is addressed in Section 2.2 of this note. We begin with some notation. We let

*Corresponding author:katia.colaneri@uniroma2.it

© The Author 2025. Published by Oxford University Press. All rights reserved. For permissions, please e-mail: journals.permissions@oup.com

- S_n be the number of susceptible individuals at time t_n;
- I_n be the number of infectious persons at time t_n who can generate new infections in the next period, i.e. $[t_n, t_{n+1})$;
- I_n^+ be the number of individuals who get infected in $[t_n, t_{n+1})$;
- I_n^- be the number of individuals who were infectious at t_n but are removed from the number of infectious people at time t_{n+1}, for instance since they recovered or, for certain diseases, are in quarantine;
- R_n be the number of so-called *removed* individuals; that is, people who are either immune or in quarantine at time t_n;
- P_n be the number of newly *reported* infections (such as positive tests) at time t_n, where we assume that testing starts at t_1;
- β_n be the *infection* or *transmission* rate, that is the average number of people that are infected in $[t_n, t_{n+1})$ by one infectious person in a population where everyone is susceptible. For convenience we also write $\Psi_n = \log(\beta_n)$.

2.1 *Models studied in Colaneri* and others *(2025)*

In this section we discuss two models for epidemics with random transmission rate. In the first model we introduce a simplified version where the state variables form an HMM and we refer to this as the case *without quarantine*. In the second model we consider the case where quarantine measures are applied, which translates into a modification in the dynamics of removed individuals through the process I_n^-. Note that the data analysis in Colaneri *and others* (2025) is based on the second model.

In the model without quarantine, the dynamics of the system are as follows. For $n = 1, 2, \ldots,$

$$\begin{cases} \Psi_n = \Psi_{n-1} + \kappa(\mu - \Psi_{n-1}) + \sigma Z_{n-1} \\ I_n = I_{n-1} + I_{n-1}^+ - I_{n-1}^- \\ R_n = R_{n-1} + I_{n-1}^- - \delta R_{n-1}\,, \end{cases} \tag{2.1}$$

where $I_n^+ \sim \text{Poisson}\big(\beta_n \frac{I_n}{N} S_n\big)$, $I_n^- = \gamma I_n$ and $Z_n \sim N(0,1)$. The reported infections satisfy $P_n \sim \text{Binomial}\left(\lfloor I_n \rfloor, q\right)$. Finally, the observations are given by the history of confirmed cases, $P_{1:n}$ for every $n = 1, 2, \ldots$.

In the model with quarantine, we have the following dynamics of the system. For $n = 1, 2, \ldots,$

$$\begin{cases} \Psi_n = \Psi_{n-1} + \kappa(\mu - \Psi_{n-1}) + \sigma Z_{n-1} \\ I_n = I_{n-1} + I^+_{n-1} - I^-_{n-1} \\ R_n = R_{n-1} + I^-_{n-1} - \delta R_{n-1}\,, \end{cases} \tag{2.2}$$

where $I^+_n \sim \text{Poisson}\big(\beta_n \frac{I_n}{N} S_n\big)$, $I^-_n = P_n + \gamma I_n$, $P_n \sim \text{Binomial}\left(\lfloor I_n \rfloor, q\right)$ and $Z_n \sim N(0, 1)$. Note that what changes here is the dynamics of I^-_n. Indeed, a person is removed from the pool of infectious people for two reasons: either if she tests positive or if she is undetected but recovers from infection.

As before, the observations are given by the history of confirmed cases, $P_{1:n}$ for every $n = 1, 2, \ldots$ and $P_n \sim \text{Binomial}\left(\lfloor I_n \rfloor, q\right)$. Finally we recall that in the model with quarantine the effective reproduction number is given by

$$\mathcal{R}_n = \frac{\beta_n}{\gamma + q - \gamma q} \frac{S_n}{N}.$$

2.2 *An extended stochastic SIR model with vaccination*

In this section we explain how the basic model of Section 2.1 (with or without quarantine) can be extended to account for vaccination. We denote by V_n the number of vaccinated individuals at t_n. We assume that only susceptible individuals are vaccinated, so that vaccinated individuals form a compartment in their own right. Hence, the following identity holds

$$S_n = N - I_n - V_n - R_n, \quad n = 0, 1, \ldots .$$

Vaccination provides partial protection against infection, and the strength of protection is governed by a parameter $\pi \in [0, 1)$. In the setup with vaccination the new infections are given, for $n = 1, 2, \ldots,$ by

$$I^+_n = I^{+,1}_n + I^{+,2}_n.$$

The process $I^{+,1}_n$ models new infections from the pool of susceptible individuals; as before, $I^{+,1}_n \sim \text{Poisson}\big(\beta_n \frac{I_n}{N} S_n\big)$. The process $I^{+,2}_n$ describes new infections from the pool of vaccinated individuals; we assume that $I^{+,2}_n \sim \text{Poisson}\big(\pi\beta_n \frac{I_n}{N} V_n\big)$ and that $I^{+,1}_n$ and $I^{+,2}_n$ are conditionally independent given I_n, V_n, R_n, β_n. Note that under these dynamics, a smaller value of the parameter π corresponds to a higher level of protection offered by vaccination. In particular, for $\pi = 0$ it holds that $I^{+,2}_n = 0$, so that vaccinated people are protected in full. This model falls into the class of *leaky vaccine models*, meaning that vaccinated

individuals experience a reduced probability of infection upon each exposure to an infectious person; the *vaccine efficacy* in this context is given by

$$\mathrm{VE} = 1 - \frac{\pi\beta_n}{\beta_n} = 1 - \pi,$$

see, for instance, Hernández-Suárez and Castillo-Chavez (2000) for details. This approach to modeling vaccination differs from the to so-called *all-or-nothing* vaccine models, in which a certain proportion of vaccinated individuals gain complete immunity, while the remainder receive no protection at all. Leaky vaccine models capture partial protection on an individual level, making them particularly suitable for diseases like Covid-19, where vaccines have been shown to reduce susceptibility and severity, but not necessarily to prevent infection entirely, especially in the case of variants such as Omicron. For a more detailed description of leaky vaccines, see Hernández-Suárez and Castillo-Chavez (2000); Edlefsen (2014); for a discussion specific to the case of COVID-19, see for example Liu and Lou (2022).

The flow variables I_n^- and P_n are as in the first model, if we consider vaccination without quarantine, or as in the second model, if one wants to model both, vaccination and quarantine. For the removed individuals, we assume that

$$R_n = R_{n-1} + I_{n-1}^- - \delta^{(1)} R_{n-1},$$

where we now use $\delta^{(1)}$ to describe the rate at which removed individuals become susceptible again. The dynamics of V_n are finally given by

$$V_n = V_{n-1} + V_{n-1}^+ - V_{n-1}^-,$$

where $V_n^+ = \nu S_n$ and $V_n^- = I_n^{+,2} + \delta^{(2)} V_n$. The parameter ν represents the *vaccination rate* and $\delta^{(2)}$ models the rate at which a vaccinated individual loses protection and gets susceptible again.

Remark 2.1 The version of the model with vaccination presented in this section is suitable for purposes of prediction. To run a statistical analysis, instead, new vaccinations V_n^+, could be observed data. Moreover, the parameters $\delta^{(2)}$ and π would be estimated upfront, via different studies. In that case, one does not need to specify the dynamics of V_n^+.

The Covid-19 pandemic demonstrated that vaccination did not fully prevent infection, particularly with the Omicron variant, but significantly reduced the likelihood of severe outcomes and hospitalization. These characteristics are consistent with leaky vaccines, which offer partial protection at individual level by low-

ering susceptibility rather than providing complete immunity. On the other hand, at the population level, such effects can be implicitly captured by our simpler model without a vaccination compartment, via the introduction of randomness into the transmission rate.

3. Statistical Methodology

In this section, we describe the statistical methodology used to handle the problem of estimating the posterior distribution of the (static) unknown parameters $\boldsymbol{\theta}$ in our model, as well as the joint posterior distribution of parameter and state variables. In particular, we adapt the nested particle filtering (NPF) algorithm of Crisan and Miguez (2018) to our setup. The NPF algorithm consists of two nested layers of particle filters: an "outer" filter, which approximates the posterior of $\boldsymbol{\theta}$ given the observations, and a set of "inner" filters, each corresponding to a sample generated in the outer layer and yielding an approximation of the posterior measure of the state process – in our case, the triple (I, R, β), or, equivalently (I, R, Ψ) – conditional on both the observations and the given sample of $\boldsymbol{\theta}$. Diversity in the parameter set is introduced via a so-called *jittering step*.

3.1 *Inner (State) Filter*

The inner layer of the nested particle filter corresponds to solving the filtering problem for known parameter values, which reduces to tracking the posterior probability distribution of the state. This task can be accomplished using standard particle (bootstrap) filtering methods, as, e.g., in Gordon *and others* (1993). To illustrate this process in the context of our specific setup, we provide the corresponding pseudocode in Algorithm 1.

3.2 *Parameter Estimation via Nested Particle Filtering*

When it comes to parameter estimation in our setup, we can distinguish two sets of parameters influencing the epidemiological system. On the one hand, we have the triple $\boldsymbol{\theta} = (\kappa, \sigma, \mu)^\top$, which governs the dynamics of the logarithmic infection rate. On the other hand, we have the rates q, γ, and δ. It is worth noting that our observation process consists only of the number of reported cases and that, due to such limitations in the nature and in the length of the observation time series, we will not be able to estimate all model parameters

Algorithm 1: Bootstrap Filter Algorithm

Input: number of continuous time units (including time 0) N^{days}, number of individuals N, values of model parameters $q, \gamma, \delta, \kappa, \sigma, \mu$, initial value of recovered people R_0, recorded observations $(p_t)_{1 \leqslant t \leqslant N^{days}}$, number of state particles M.

Initialization

```
for m = 1 to M
    Sample (i_0^(m), r_0^(m), ψ_0^(m)) from the prior distribution        // See Appendix B and C
end
```

Recursive step (t**-th iteration, where** $t \geqslant 1$)

Propagate

for $m = 1$ **to** M

Draw $\bar{\psi}_t^{(m)}$ conditional on $\psi_{t-1}^{(m)}$

Draw $\left(\bar{i}_t^{(m)}, \bar{r}_t^{(m)}\right)$ conditional on $\left(i_{t-1}^{(m)}, r_{t-1}^{(m)}\right)$ in the no-quarantine model resp.

conditional on $\left(i_{t-1}^{(m)}, r_{t-1}^{(m)}\right)$ and the observation p_{t-1} in the model with quarantine

`// See updating schemes (2.1) or (2.2)`

end

Compute normalized likelihood weights

for $m = 1$ **to** M

Let $a_t^{(m)} = \binom{\lfloor \bar{i}_t^{(m)} \rfloor}{p_t} q^{p_t} (1-q)^{\lfloor \bar{i}_t^{(m)} \rfloor - p_t}$

Compute $\bar{w}_t^{(m)} = \frac{a_t^{(m)}}{\sum_{m=1}^{M} a_t^{(m)}}$.

end

Resample with replacement

for $m = 1$ **to** M

$\left(i_t^{(m)}, r_t^{(m)}, \psi_t^{(m)}\right) = \left(\bar{i}_t^{(j)}, \bar{r}_t^{(j)}, \bar{\psi}_t^{(j)}\right)$ with probability $\bar{w}_t^{(j)}$, $j \in \{1, \ldots, M\}$

end

Output: Approximate state posterior distribution given observations and parameters

with reasonable accuracy and we will thus resort to fixing the rates q, γ and δ exogenously. That is, we assume that the parameters q, γ, and δ are derived from other data (for instance, different medical data, sewage data, as well as the results of other statistical studies) and hence represent a fixed input of our model.

Nested Particle Filtering Algorithm Next, we provide a schematic description of the algorithm and the pseudocode in Algorithm 2. For further computational details, the reader is referred to Appendix A; moreover, inputs and prior choices for the simulation (resp. the data analysis) are given in Appendix B (resp. Appendix C).

In the initialization step K particles for the parameter vector are drawn from a prior distribution, say μ_0, and, for each of these particles, M particles for the state vector are drawn from a distribution π_0. To

simplify the notation, in the sequel we denote the output of the NPF algorithm for every iteration $t \geqslant 1$ as $\theta_t^{(k)} = (\kappa_t^{(k)}, \sigma_t^{(k)}, \mu_t^{(k)})$ and $x_t^{(k,m)} = (i_t^{(k,m)}, r_t^{(k,m)}, \psi_t^{(k,m)})$ for all $k = 1, \ldots, K$, and $m = 1, \ldots, M$.

In each iteration, the parameter particles are first agitated, or *jittered*, using a truncated Gaussian kernel with mean corresponding to the parameter estimates at the previous time point and fixed variance (denoted later by ϵ_κ, ϵ_σ and ϵ_μ). In this way, all particles are subjected to a small perturbation, restoring their diversity. Then, for each of these parameter sets, the state particles are propagated using the model dynamics (2.1) with the jittered parameter values. Next, the likelihood of each particle is computed using the new information (i.e., the observed positive tests p_t), and state particles are resampled with replacement as in a standard particle filter. Note that, at the end in the iteration t where the number of positive test $p_t = p_n$ is used to compute the likelihood, we obtain an approximation of the posterior distribution for state variables I_n, R_n and Ψ_{n-1} (this is due to the way in which the process Ψ influences the observations: in particular, Ψ_{n-1} influences I_{n-1}^+ and thus, indirectly, I_n and P_n). Finally, parameter particles are resampled with replacement according to their likelihood, which then provides an approximation of the posterior distribution of the parameters. A basic assumption of the NPF algorithm is that the parameter space is a compact set and the conditional probability distribution of the observations is bounded uniformly over that set. These assumptions are satisfied in our case by the choice of the initial distribution and the jittering kernel, see Appendix B and Appendix C.

4. Simulation results

The goal of this section is to test the nested particle filter approach described in Section 3 on simulated data that mimic typical features of Austrian Covid-19 data. In Section 4.3 we moreover compare our method to the iterated filtering approach of Ionides *and others* (2015).

4.1 *Results on simulated infection data*

We work in the context of the model with quarantine restrictions, with the dynamics given by equations (2.2) (see also Section 2.2 of Colaneri *and others* (2025)). The *true* parameters used to generate state and observation time series are given in Appendix B. We take the probability of a positive test, the average duration of the illness, and the average natural immunity period (i.e. $q, 1/\gamma, 1/\delta$) as given and we fix them

Algorithm 2: Nested Particle Filter Algorithm

Input: number of continuous time units (including time 0) N^{days}, number of individuals N, values of fixed parameters q, γ, δ, initial value of recovered people R_0, recorded observations $(p_t)_{1 \leqslant t \leqslant N^{days}}$, number of state particles M and number of parameter particles K, jittering hyperparameters.

Initialization

for $k = 1$ **to** K

  Sample $\theta_0^{(k)}$ from the prior distribution

  for $m = 1$ **to** M

    Sample $x_0^{(k,m)}$ from the prior distribution `// See Appendix B and C.`

  end

end

Recursive step **(t-th iteration, where $t \geqslant 1$)**

Let, for $t \geqslant 1$, $(\theta_{t-1}^{(k)}, x_{t-1}^{(k,m)})$ be the set of available samples at time $t-1$

for $k = 1$ **to** K

  Draw $\bar{\theta}_t^{(k)} = (\bar{\kappa}_t^{(k)}, \bar{\sigma}_t^{(k)}, \bar{\mu}_t^{(k)})$ from $f_K^{\theta_{n-1}^{(k)}}(\mathrm{d}\theta)$ `//` f_K `is the jittering kernel.`

end

for $m = 1$ **to** M

  Inner filter

    Propagate

      Draw $\bar{\psi}_t^{(k,m)}$ conditional on $\psi_{t-1}^{(k,m)}$

      Draw $(\bar{i}_t^{(k,m)}, \bar{r}_t^{(k,m)})$ conditional on $(i_{t-1}^{(k,m)}, r_{t-1}^{(k,m)})$, and if needed p_{t-1} `// See updating schemes (2.1) or (2.2)`

    Compute normalized likelihood weights

      Let $a_t^{(k,m)} = \binom{\lfloor \bar{i}_t^{(k,m)} \rfloor}{p_t} q^{p_t} (1-q)^{\lfloor \bar{i}_t^{(k,m)} \rfloor - p_t}$

      Compute $\bar{w}_t^{(k,m)} = \frac{a_t^{(k,m)}}{\sum_{m=1}^{M} a_t^{(k,m)}}$.

    Resample with replacement

      Let $\tilde{x}_t^{(k,m)} = \bar{x}_t^{(k,j)}$ with probability $\bar{w}_t^{(k,j)}$, $j \in \{1, \ldots, M\}$

end

Compute normalized likelihood weights

  Compute $\bar{w}_t^{(k)} = \frac{\sum_{m=1}^{M} a_t^{(k,m)}}{\sum_{k=1}^{K} \sum_{m=1}^{M} a_t^{(k,m)}}$

Resample with replacement

  Let $\left(\theta_t^{(k)}, \left\{x_t^{(k,m)}\right\}_{1 \leqslant m \leqslant M}\right) = \left(\bar{\theta}_t^{(l)}, \left\{\tilde{x}_t^{(k,m)}\right\}_{1 \leqslant m \leqslant M}\right)$ with probability $\bar{w}_t^{(l)}$, $l \in \{1, \ldots, K\}$.

Output: approximate parameter posterior distribution with $\frac{1}{K} \sum_{k=1}^{K} \delta_{\theta_t^{(k)}}$ for each $t = 1, \ldots, N^{days}$,

where δ_θ denotes the Dirac measure at θ .

consistently with the values reported by e.g. the Austrian Ministry of Health, see Richter *and others* (2020).

We run the simulation for a period of two years (731 days), which is roughly consistent with the length of the real time series used for the application in Section 4 of Colaneri *and others* (2025). Figure 1 depicts one simulated trajectory of the observation sequence P_n, for $n = 1, \ldots, 731$, which is used as a basis for our analysis. This path shows qualitative properties that are similar to real Covid-19 infection data: for instance, our model naturally generates waves of infections. Note that, towards the end of the simulation period, the number of positive tests becomes low. Below we will see that this affects the accuracy of the estimation of the infection rate and the effective reproduction number (see Figure 2).

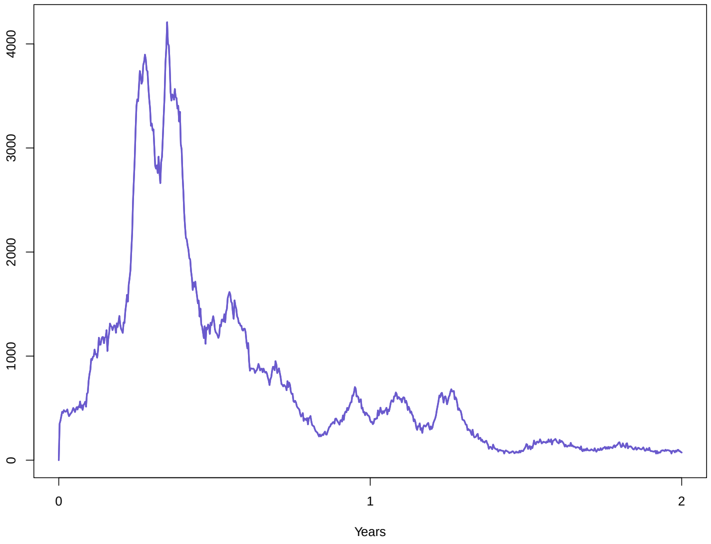

Figure 1. Simulated trajectory of the number of positive tests (observation process).

We used the NPF approach described in Section 3 to estimate the number of infections, the infection rate $(\beta_n)_{n\geqslant 1}$, the reproduction number $(\mathcal{R}_n)_{n\geqslant 1}$ and the model parameters. The results of this analysis are discussed next.

Reproduction rate. Figure 2 displays the true (in black) and filtered (in magenta) trajectory of the effective reproduction number $(\mathcal{R}_n)_{n\geqslant 1}$. The grey area provides a credible interval. We recommend the reader to use a color (or screen) version of this plot for a better understanding. This plot suggests that the true trajectory exhibits higher variance than the filtered one: the filter generally captures the trend of the state process quite well, but it is not able to detect small movements in a short time interval. Moreover, as mentioned previously, the number of positive tests is quite low towards the end of the simulation period, and thus, as

the observation process becomes less informative, the accuracy of the filter decreases. Moreover, we carried out robustness checks to ensure that small errors in the values of the parameters q and γ (which are specified outside of the estimation procedure) do not affect our estimates too strongly. These robustness checks yielded good results; details are omitted.

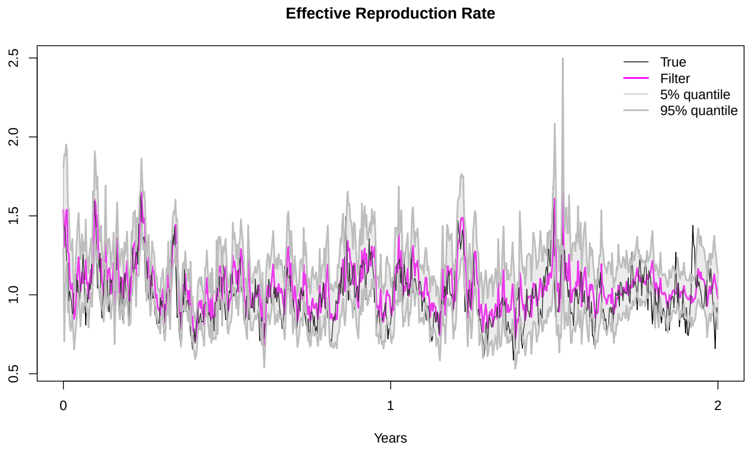

Figure 2. True (black) and filtered (magenta) trajectory of the effective reproduction number. The gray area represents a credible interval.

Parameters. Next, we discuss the estimation of the three unknown model parameters κ, σ, and μ that govern the dynamics of the transmission rate β_n. Their posterior distributions are obtained through the NPF algorithm and visualized in Figure 3. In each panel, the black line corresponds to the true value of the parameter, the two blue lines to the 5%- and 95%-quantile of the posterior distribution (as obtained by the nested particle filter) and the red line to the mean.

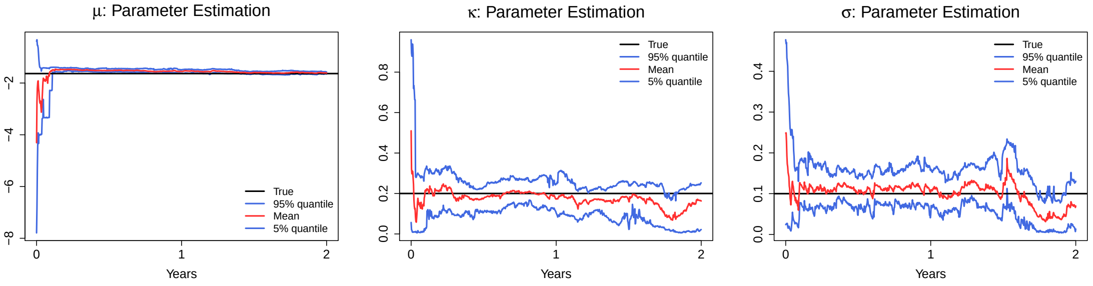

Figure 3. Posterior estimates for μ, κ, and σ. The black line corresponds to the true value of the parameter, the two blue lines to the 5%- and 95%-quantile of the posterior distribution (as obtained by the nested particle filter), and the red line to its mean.

We observe that the posterior-mean estimate of μ quickly settles around the true value, while κ and σ seem to be more difficult to estimate. We attribute this difficulty to a couple of reasons. First, we have relatively few observations, corresponding to two years. We decided to run our algorithm for two years

only to be in line with the amount of data we used in our case study for the Austrian Covid-19 data, see (Colaneri *and others*, 2025, Section 4). Second, we considered a quite small support for these parameters, within which it might be more challenging to further discriminate between plausible values in a short period. Nevertheless, the algorithm can detect parameter magnitudes quite rapidly. Moreover, note that there might be a 'countervailing' effect at play between posterior-mean estimates for σ and κ, since the long-run variance of Ψ_n is $\sigma^2/2\kappa$, and their distinct impact on state and observations might be decoupled by the algorithm only over a longer time horizon.

Mean Relative Error over independent algorithm runs. In Figure 4 we plot the mean relative errors between true values and posterior-mean estimates on a logarithmic scale, averaged over the 50 independent runs of the algorithm on the dataset from Figure 1. This serves to gauge the robustness of our methodology with respect to sampling noise in the NPF. These plots appear consistent with our considerations: (i) estimation errors for μ decrease quickly, as expected; (ii) mean relative errors for the parameters κ and σ support the difficulty in the estimation of these parameters; (iii) one can also compare the behavior of parameter estimation errors for κ and σ with those for the ratio $\sigma^2/2\kappa$. In interpreting these plots, however, one should bear in mind that the true value of the ratio $\sigma^2/2\kappa$ is very small (equal to 0.025) so that considering relative errors, instead of absolute ones, might exaggerate the variability of the estimation error.

4.2 *Performance for different simulated data sets*

Using the parameters and model specifications from Section 4.1, we generated 50 paths of the epidemiological system and ran the NPF algorithm on each time series of observations. This experiment allows us to gauge the variability in the algorithm performance with respect to the specific realization of the epidemiological system. In Figure 5 we plot histograms of the posterior-mean estimates for parameters μ, κ, σ, and the ratio $\sigma^2/2\kappa$, both halfway through the observation period and at terminal time. We can draw similar considerations as in the previous section. In particular, μ is the easiest parameter to estimate, and the algorithm performance appears satisfactory overall.

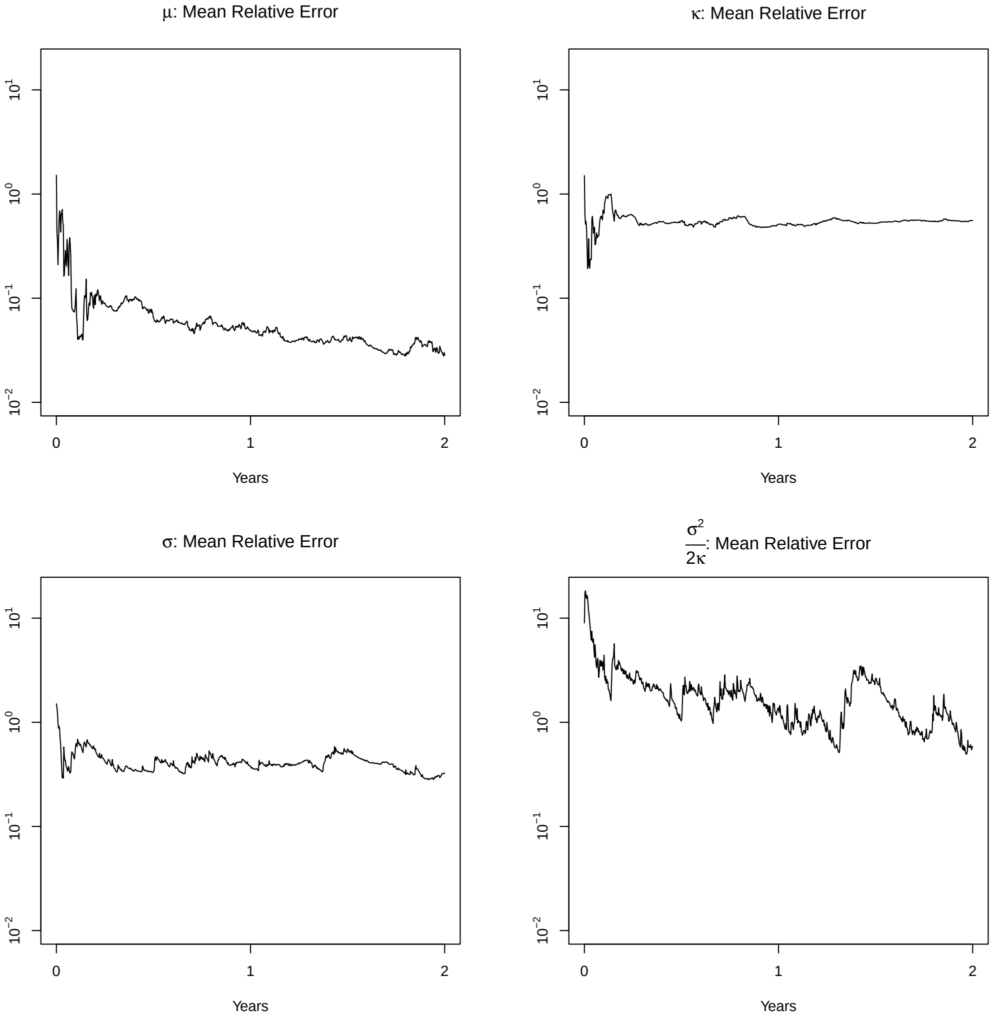

Figure 4. Mean relative errors for μ (top left panel), κ (top right panel), σ (bottom left panel) and $\sigma^2/2\kappa$ (bottom right panel), on a logarithmic scale for the data set from Figure 1 for 50 independent runs of the NPF algorithm.

4.3 *Comparison with Iterated Filtering*

Finally, we discuss a comparison of our methodology to the iterated filtering (IF) algorithm of Ionides *and others* (2015). The two approaches are quite different in spirit and, crucially, only NPF is recursive. We use the R package pomp (see King *and others* (2016)) to simulate one realization of the epidemiological system and to run the IF algorithm on the time series of observations using function mif2. We therefore considered a version of the model without quarantine, so that we are in a classical HMM setup. Model parameters and the specifications for the NPF were taken as in Section 4.1. For IF, we use 100 iterations with 500 particles, as well as starting and perturbation values comparable to the setup of NPF: in particular, we use as starting value for each unknown parameter the midpoint of its corresponding NPF prior, and a perturbation of 0.02

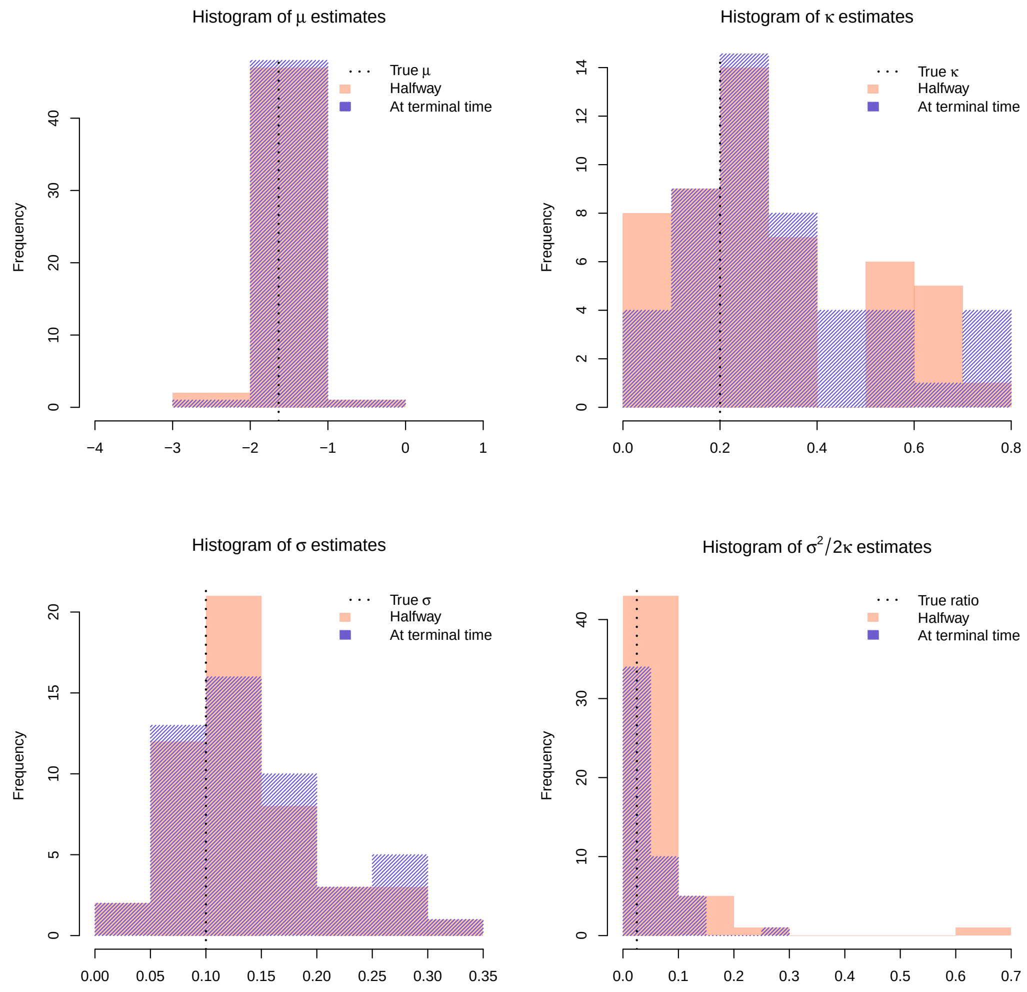

Figure 5. Histograms of posterior-mean estimates for μ, κ, σ and $\sigma^2/2\kappa$ for different data sets, both halfway trough the observation period (coral) and at terminal time (blue).

for all parameters with geometric cooling of base $\alpha = 0.5$, to achieve, at the 100th iteration of the algorithm, a perturbation comparable to the standard deviation of the jittering kernel in NPF. The parameter estimation results for the two methods are depicted in the top and middle panels of Figure 6. We see that the parameter estimates provided by the two methods are relatively close.

Note that the application of IF in our context should be preceded by a comprehensive simulation study that assesses the performance of IF for different hyperparameter choices (and starting points) for fine-tuning the method. To illustrate why this is important, we refer to the bottom panel of Figure 6. There we set a slightly 'slower' cooling schedule by changing the base to $\alpha = 0.55$ (keeping everything else equal), and keeping the same seed as before. It turns out that already such a small variation in α, leads to quite different behavior of the estimates, in particular for κ.

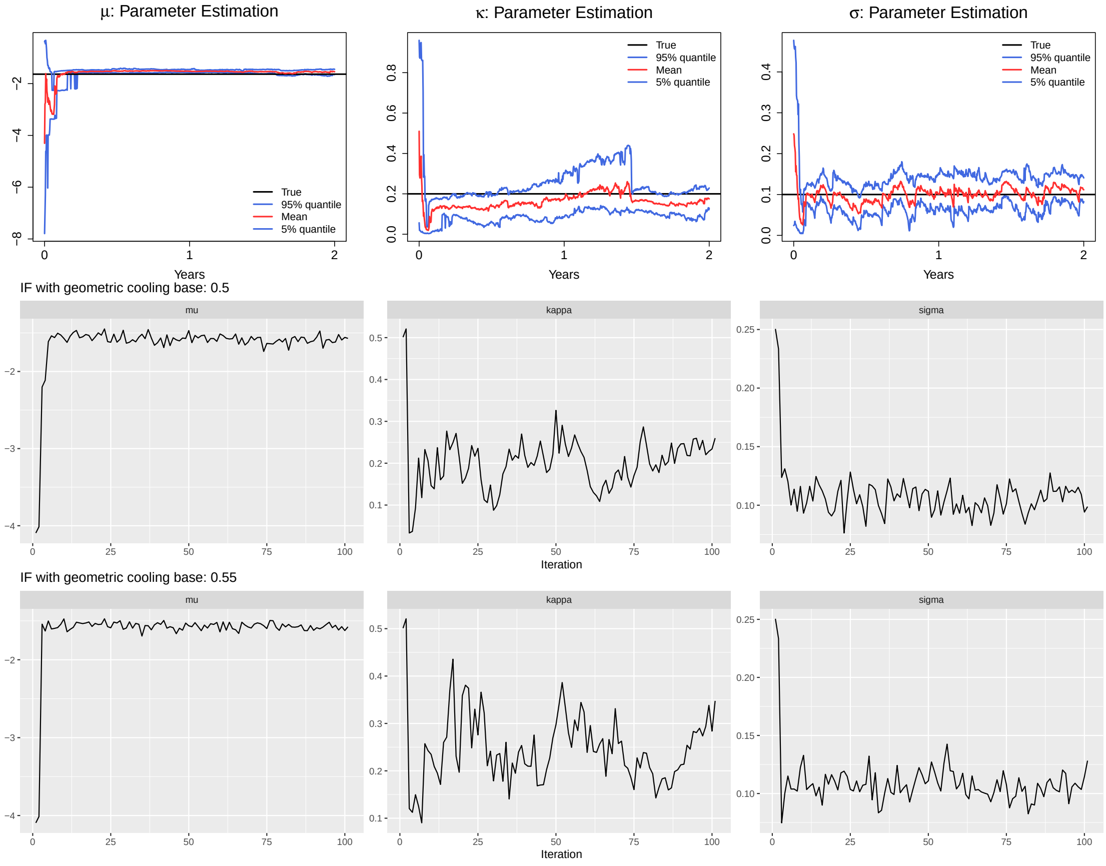

Figure 6. Posterior estimates for μ, κ, and σ using NPF (upper panel), and final parameter estimates using IF with cooling base $\alpha = 0.5$ (middle panel) and $\alpha = 0.55$ (bottom panel).

APPENDIX

A. Details on nested particle filtering

A.1 *Non-Integer Quantities.*

Note that some quantities in the models described by (2.1) and (2.2) are not integers due to the presence of rates $\gamma > 0$ and $\delta > 0$, while others (e.g. P and I^+) are nonnegative integers by construction. In particular, it might be that the process I, i.e., the number of infectious people, gets below one (or even becomes negative) for some particles at some point in time. To avoid this issue, we artificially set $I_n = 0$, whenever a particle is propagated to a negative value. Notice that P_n could be a positive integer even if for some particles $\lfloor I_n \rfloor = 0$. In this case, the likelihood of the observation given the particle is zer,o and hence the particle is eliminated in the resampling.

B. Inputs for the simulation study

Simulation parameters (true values) and other inputs. We consider the following specifications: $q = 10\%$, $\gamma = 1/10$, $\delta = 1/200 = 0.05$, $\kappa = 0.2$, $\sigma = 0.1$, $\mu = \log(\gamma + q - \gamma q) - \frac{\sigma^2}{2\kappa} \approx -1.63$. We take number of individuals $N = 8.917 \cdot 10^6$, number of days $N^{days} = 731$ (including time 0), a time step: $\Delta = t_n - t_{n-1} = 1$ day and initial value for $R_0 = 0$.

Settings for the Nested Particle Filter

- Prior for Ψ: normal with mean μ and standard deviation 0.175.
- Prior for I: Gamma (to ensure non-negativity) with mean 3000 and variance 15000.
- Prior for κ, σ and μ: uniform over 1% and 500% of true values.
- Number of particles in the state space $M = 500$ and in the parameter space $K = 500$.
- Variance of the jittering kernels $\epsilon_\kappa = \epsilon_\mu = \epsilon_\sigma = 5K^{-2}$.

C. Inputs for the real data analysis

Data characteristics

- Time period: from May 1, 2020, to June 15, 2022.
- Total number of individuals: $N = 9.028 \cdot 10^6$ (i.e. population of Austria).
- Observations P: 7-day rolling average of confirmed cases.

Fixed parameters and other inputs. We take $q = 10\%$, $\gamma = 1/10$, $\delta = 1/200 = 0.05$, number of days: $N^{days} = 776$ (including time 0), time step: $\Delta = t_n - t_{n-1} = 1$ day and $R_0 = 0$.

Settings for the Nested Particle Filter

- Prior for Ψ: normal with mean $\log(\gamma + q - \gamma q) \approx -1.66$ and standard deviation 0.1.
- Prior for I: Gamma (to ensure non-negativity) with mean 470 and variance 2350, where the mean corresponds to $\bar{p}/q$ and $\bar{p} = 47$ to the 7-day rolling average of confirmed cases on the day preceding the start of our data analysis (i.e. on April 30, 2022).

- Prior for κ: uniform over $[0.01, 1]$. This prior is chosen to be uninformative.
- Prior for σ: uniform over $[0.01, 1]$ (uninformative).
- Prior for μ: uniform over $[-4, -0.01]$ (uninformative).
- Number of particles in the state space $M = 500$ and in the parameter space $K = 500$.
- Variance of the jittering kernels $\epsilon_\kappa = \epsilon_\mu = \epsilon_\sigma = 5K^{-2}$.

References

Colaneri, K., Damian, C. and Frey, R. (2025). A filtering approach for statistical inference in a stochastic SIR model with an application to COVID-19 data. *Biostatistics*, DOI: `10.1093/biostatistics/kxaf036`.

Crisan, D. and Miguez, J. (2018). Nested particle filters for online parameter estimation in discrete-time state space Markov models. *Bernoulli* **24**, 3039–3086.

Edlefsen, P. (2014). Leaky vaccines protect highly exposed recipients at a lower rate: implications for vaccine efficacy estimation and sieve analysis. *Computational and Mathematical Methods in Medicine* **1**, 813789 (12 pages).

Gordon, N., Salmond, D. and Smith, A. (1993). Novel approach to nonlinear/ non-Gaussian Bayesian state estimation. *IEE Proceedings F (Radar and Signal Processing)* **140**(2), 107–113.

Hernández-Suárez, C. and Castillo-Chavez, C. (2000). Urn models and vaccine efficacy estimation. *Statistics in medicine* **19**(6), 827–835.

R Core Team. (2022). *R: A Language and Environment for Statistical Computing*. R Foundation for Statistical Computing, Vienna, Austria.

Richter, L., Schmidt, D. and Stadlober, E. (2020). Methodenbeschreibung für die Schätzung von epidemiologischen Parametern des COVID 19 Ausbruchs Österreich. Working paper, AGES, available from `https://www.ages.at/`.

King, A., Nguyen, D. and Ionides, E. (2016). Statistical Inference for Partially Observed Markov Processes via the R Package pomp *Journal of Statistical Software* **69**(12), 1–43.

Ionides, E., Nguyen, D., Atchadé, Y., Stoev, S. and King, A. (2015). Inference for dynamic and latent variable models via iterated, perturbed Bayes maps *Proceedings of the National Academy of Sciences* **112**(3), 719–724.

Liu, K. and Lou, Y. (2022). Optimizing COVID-19 vaccination programs during vaccine shortages *Infectious Disease Modelling* **7**(1), 286–298.
